# Prevalence of *Fasciola hepatica* infection in slaughtered sheep from Northwest Tunisia and its risk factors: Association with gastrointestinal helminths infection and anaemia

**DOI:** 10.1002/vms3.1575

**Published:** 2024-08-27

**Authors:** Ines Hammami, Yosra Amdouni, Rihab Romdhane, Limam Sassi, Nadia Farhat, Mourad Rekik, Mohamed Gharbi

**Affiliations:** ^1^ Laboratoire de Parasitologie Univ Manouba École Nationale de Médecine Vétérinaire de Sidi Thabet Sidi Thabet Tunisia; ^2^ Faculty of Sciences of Tunis University of Tunis El Manar Manar II Tunis Tunisia; ^3^ Circonscription de la production animale Bizerte Tunisia; ^4^ International Center for Agricultural Research in the Dry Areas (ICARDA) Ariana Jordan

**Keywords:** Bile, coprology, fasciolosis, gastrointestinal helminths, sheep, Tunisia

## Abstract

We investigated herein the prevalence of *Fasciola hepatica* infection in sheep at Sejnane slaughterhouse, governorate of Bizerte, Northwest of Tunisia, using three different diagnostic techniques (liver dissection, bile examination, and coprology). Faeces, liver, gall bladder as well as blood samples were collected from 603 slaughtered sheep in two seasons: winter and summer. Faecal egg counts of *F. hepatica* were estimated using sedimentation technique. Livers were examined for the presence of flukes, and bile collected from gall bladder was examined by sedimentation technique for the presence of *F*. *hepatica* eggs. Faecal egg counts of gastrointestinal helminths were estimated using flotation followed by the McMaster technique. Blood samples were used to estimate blood cell count (RBC) (×10^6^/mL), haemoglobin (Hb) (g/dL), and haematocrit (Ht) (%) levels. A total of 1714 *F*. *hepatica* flukes were collected from 68 infected livers, the number of flukes per sheep ranged between naught and 195. Bile examination (16.78% ± 1.83; 51/310) showed the higher infection prevalence, followed by liver dissection (11.28% ± 1.17; 68/603) and coprology (9.12% ± 1.08; 55/603) (*p* = 0.015). Infection prevalences were significantly higher in young sheep aged of less than 1 year (8.13% ± 1.22; 49/498), in cross‐bred sheep (10.61% ± 1.39%; 64/478), and in summer (7.13% ± 1.82; 43/293) (*p* < 0.05). There was no significant difference in infection prevalence by gastrointestinal helminths in *F*. *hepatica*‐infected and *F*. *hepatica‐*non‐infected animals (*p* > 0.05). The overall prevalence of *F*. *hepatica*‐infected anaemic sheep was higher (22.73% ± 4.47; 20/88) than *F*. *hepatica*‐non‐infected anaemic sheep (*p* < 0.05). *Fasciola hepatica* infection is frequent in sheep from Sejnane representing hence an important constraint for the development of the sheep industry in this region. Therefore, it is necessary to establish and implement a specific control programme to reduce fasciolosis infection risks including animal owners’ education.

## Graphical abstract

Fasciolosis is one of the main parasitic infections of sheep in northwestern Tunisia, where bile examination sensitivity was higher than both liver inspection and coprology. Infected anaemic sheep prevalence was higher than that of non‐infected anaemic sheep.

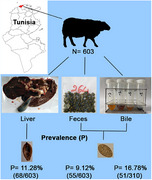

## 1 INTRODUCTION

Fasciolosis is a hepatobiliary parasitic zoonotic disease common to various herbivorous mammals particularly cattle and sheep. Humans can be infected by this parasite (Evack et al., 2020; Mas‐Coma et al., 2009; Omar et al., 2021; Sabourin et al., 2018). Fasciolosis is caused by two digenean, namely *Fasciola hepatica* and *Fasciola gigantica* (Itagaki et al., 2022). Both species have a worldwide distribution: *F*. *hepatica* is common primarily in temperate zones, including parts of Africa, Europe, Middle East, Asia, Latin America, Caribbean, and Oceania, while *F*. *gigantica* predominates in African and Asiatic tropical regions (Afshan et al., 2014; Mas‐Coma et al., 2005; Meerkhan & Razak, 2014; Periago et al., 2008). Moreover, the two *Fasciola* species could coexist in regions where environmental conditions allow the development of their intermediate hosts mainly in North Africa, East African highlands, and Asia (Malatji et al., 2019; Mas‐Coma et al., 2009), with some hybrid populations present in Egypt, Iran, Vietnam, and Japan (Itagaki et al., 2005; Ichikawa & Itagaki, 2012; Anh et al., 2018; Itagaki et al., 2022). *Fasciola* spp. life cycle involves an intermediate host, a freshwater snail belonging to the family of *Lymnaeidae* with *Galba* (*Lymnaea*) *truncatula*, a major host of *F*. *hepatica*, and *Radix* genera as primary hosts of *F*. *gigantica* (Correa et al., 2010; Mas‐Coma et al., 2019; Radostits et al., 2007; Torgerson & Claxton, 1999). Furthermore, low temperatures, humid climate, swamp zones, and soil type favour a higher *Fasciola* infection rate (Zhuo et al., 2023; Nguyen et al., 2021; Shykat et al., 2022).

Fasciolosis is transmitted to animals during grazing and to humans by eating crude vegetables contaminated by metacercariae (Mas‐Coma et al., 2005; Usip et al., 2014). Previous works reported that in the world, approximately 17 and 180 million humans are infected and at risk of infection by *Fasciola*, respectively (Nyindo & Lukambagire, 2015). The liver fluke infection affects animals’ productivity and welfare causing considerable financial losses worldwide estimated to 3.2 US$ billion per year (Mehmood et al., 2017), due to reduced milk yields and fertility, decrease in growth rates, poor quality of carcass, liver condemnation, and mortality (Hayward et al., 2021; Mazeri et al., 2017; Opio et al., 2021; Tsega et al., 2015). *Fasciola* infection diagnosis in bovine is based on the detection of flukes in the liver bile ducts during *post‐mortem* examination, coproscopy, and antibody screening in either serum or milk (Charlier et al., 2008; Nasreldin & Zaki, 2020; Simo et al., 2020). The *post‐mortem* examination represents the most useful technique in slaughterhouses for monitoring this infection (Birhanu et al., 2015; Çelik & Aslan Çelik, 2018; Chaouadi et al., 2019).

In Tunisia, livestock breeding plays an essential role in the national economy through the wool, milk, meat, and leather production. It is also considered a primary source of income for small farmers. However, the Tunisian livestock population suffers from several parasitic infections such as gastrointestinal parasites (Akkari et al., 2012), tick‐borne infections (Khbou et al., 2021), and fasciolosis. The latter is highly prevalent in sheep with infection prevalence rates reaching 70% in tracer lambs in Sejnane region, Northwest Tunisia, the most endemic region in the country due to its humid climate and high sheep population density (Akkari et al., 2011). In other endemic regions, the prevalence was lower, 35% and 44% in the regions of Gafsa and Tozeur, Southwest Tunisia, respectively (Ayadi et al., 1997; Hammami et al., 2005). To our knowledge, there are neither standardised control programmes adapted to the regional epidemiological context of fasciolosis nor owners’ education programme in Tunisia (Akkari et al., 2011).

The main object of the present study was to investigate *Fasciola* infection in a large number of sheep slaughtered at Sejnane slaughterhouse, Northwest of Tunisia, using for the first time three different diagnostic techniques (liver dissection, bile examination, and coprology) and assessing some infection risk factors. Moreover, to evaluate the association between *Fasciola* and gastrointestinal parasites infections and the status of anaemia in *Fasciola*‐infected and *Fasciola*‐non‐infected sheep.

## 2 MATERIALS AND METHODS

### 2.1 Study area

**FIGURE 1:**
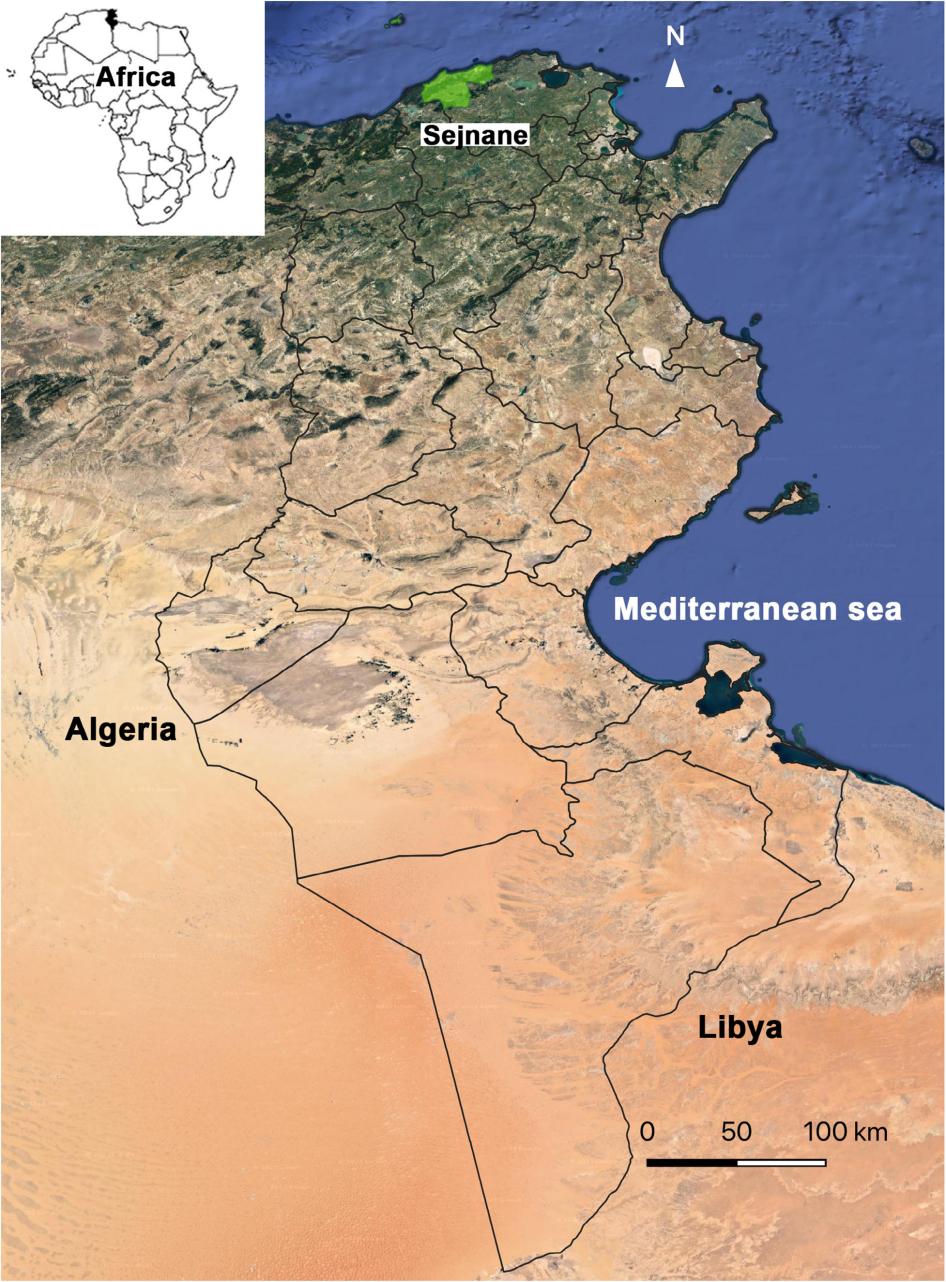
Map of Tunisia showing the sampling region.

The present study was carried out in the region of Sejnane, district of Bizerte, Northwest of Tunisia (37°06ʹ N; 09°14ʹ E) (Figure 1). Sejnane belongs to the humid bioclimatic stratus; it is characterised by a Mediterranean climate with a mean annual rainfall of 507 mm. The average minimum and maximum temperatures of 10.7 and 30°C are recorded in January and July 10.7 and 30°C, respectively (National Institute of Meteorology, 2021). Livestock breeding is the principal activity in this locality, where the sheep population is the most abundant ruminant species (20,000 female units), compared to cattle and goats, counting around 5250 and 3600 female units, respectively (Ministry of Agriculture, Hydraulic Resources, & Sea Fishing, 2022).

### 2.2 Sample collection

A total of 603 sheep samples were randomly collected between July 2020 and March 2021 in the local slaughterhouse of Sejnane. Firstly, the age of the animals was estimated by dental examination (Vandiest, 2004), blood samples were taken from each sheep before slaughter by puncture of the jugular vein using sterile Ethylenediamine tetraacetic acid (EDTA) tubes, and faeces were subsequently taken from the sheep's rectum. After slaughter, the liver and gall bladder of each animal were taken and placed in identified sterile bags.

All samples were transported, in isothermal coolers, to the Laboratory of Parasitology at the National School of Veterinary Medicine of Sidi Thabet, Tunisia, where they were analysed.

### 2.3 Animals selected

Sheep sampled belonged to four breeds: 79.27% (478/603) cross‐bred, 11.44% (69/603) Noire de Thibar, 8.62% (52/603) Queue Fine de l'Ouest, and 0.66% (4/603) Barbarine. The majority were females, representing 94.86% (572/603) of the total sampled animals. Of these sheep, 82.59% (498/603) were less than 1 year old, 13.76% (83/603) were between 1 and 3 years old, and 3.65% (22/603) were over 3 years old. The sheep were sampled over two seasons, 48.59% (293/603) in summer and 51.41% (310/603) in winter.

## 3 LABORATORY TESTS

### 3.1 Liver dissection and fluke's collection

At the beginning, an incision along the bile ducts was performed for each liver using a sterile disposable blade, then the hepatic parenchyma, bile ducts, and gall bladder were examined for the presence of immature and adult *F. hepatica*. All present liver flukes were collected, placed in identified vials, and stored in 70% ethanol.

### 3.2 Bile examination

Due to the working conditions, it was only possible to examine 304 bile samples from 603 animals. In fact, during sampling, around 50% of gall bladders were discarded by slaughterhouse workers. For each gall bladder, the bile was poured into a stem glass and allowed to sediment for 15 min. The sediment was aspirated using a pipette, placed in a Petri dish, and stained with 1 mL of 1 p. 1000 methylene blue. The solution was then examined under a stereomicroscope at ×100 magnification to count the number of *F*. *hepatica* eggs (Simo et al., 2020).

### 3.3 Coprological examination

For all faeces samples, faecal oocyst of *Eimeria* spp. and egg qualification of *Monie* *zia* spp., *Nematodirus* spp., and strongyle type egg (STE) were estimated using 5 g of faeces mixed with 75 mL of a dense solution by the flotation technique, and then the quantification of oocysts and eggs in positive samples was estimated by the McMaster technique (Zajac et al., 2012). Each compartment of the McMaster slide has a volume equal to 0.15 mL, and since the solution is diluted fifteen times, the number of oocysts and eggs counted corresponds to the number of oocysts and eggs present in one‐hundredth of 1 g of faeces. Thus, the result was multiplied by a correction factor of 100.

The presence of *F. hepatica* eggs was checked by the sedimentation‐centrifugation technique as described by Simo et al. (2020). Briefly, 5 g of stool collected from each animal were mixed with 75 mL of distilled water, filtered, then centrifuged at 1500 rpm for 15 min. After centrifugation, 2 mL of pellet was collected and stained by adding 1 mL of 1 p. 1000 methylene blue. The solution was transferred to a Petri dish to estimate the number of *F. hepatica* eggs under a stereomicroscope at ×100 magnifications.

### 3.4 Haematological analyses

Blood samples were collected from each sheep before slaughtering by puncture of the jugular vein using sterile EDTA tubes under the supervision of an officially certified veterinarian by the Tunisian Ministry of Agriculture and the Tunisian National Council of Veterinarians. Blood collected was used to estimate haematological parameters, mainly red blood cell count (RBC) (×10^6^/mL), haemoglobin (Hb) (g/dL), and haematocrit (Ht) (%). Haematological parameters were measured using an auto‐haematology analyser BC‐2800Vet (Shenzen Mindray Bio‐Medical Electronics Co., Ltd.).

Sheep were considered anaemic when the three parameters, RBC, Hb, and Ht, were below the minimum threshold values 9 × 10^12^/L, 9 g/dL, and 27%, respectively (Newcomer et al., 2021).

### 3.5 Data analysis

All the data were compiled in an Excel spreadsheet. Parasitological indicators were estimated as indicated in Table 1 (Margolis et al., 1982).

**TABLE 1:** Parasitological indicators according to sample type

Sample type			
Parasitological indicators	Liver	Faeces	Bile
**Prevalence**	100 × number of infected sheep/number of examined sheep	100 × number of infected sheep/number of examined sheep	100 × number of infected sheep/number of examined sheep
**Intensity**	Number of collected flukes in livers/number of infected sheep	Number of eggs per gram of faeces/number of infected sheep	Number of eggs per millilitre of bile/number of infected sheep
**Abundance**	Number of collected flukes in livers/number of examined sheep	Number of eggs per gram of faeces/number of examined sheep	Number of eggs per millilitre of bile/number of examined sheep

The infection prevalences of *F. hepatica* according to breed, gender, age, and season were compared using the χ and Fisher exact test. *Fasciola hepatica* infection intensity and abundance according to breed, gender, age, and season were compared using Mann–Whitney and Kruskal‐Wallis. All tests were considered significant at the 5% threshold (Schwartz, 1993).

The concordance coefficient between liver dissection/coprological examination, liver dissection/bile examination, and coprological examination/bile examination was estimated with the Kappa test (Newman, 2010). The classification of Landis and Koch (1977) for the interpretation of the Kappa values was used as follows: K < 0 no agreement, 0 < K < 0.2 slight agreement, 0.21<K<0.40 fair agreement, 0.41<K<0.60 moderate agreement, 0.61<K<0.80 substantial agreement, and 0.81<K<1 is considered almost perfect.

The association between *F. hepatica* and gastrointestinal helminths infection was determined on the basis of coprological findings, that is, the presence or absence of oocysts or eggs of each parasite in the faeces of each animal examined.

The infection prevalences of *F. hepatica* according to the presence of gastrointestinal parasites were compared using theχ and Fisher exact test. *Fasciola hepatica* infection intensity and abundance according to the presence of gastrointestinal parasites were compared using Mann‐Whitney and Kruskal‐Wallis tests. All tests were considered significant at the 5% threshold (Schwartz, 1993).

Totals, percentages, and standard deviations were calculated using Excel (version 10, Microsoft) and statistical tests were performed using SPSS (version 26, IBM).

## 4 RESULTS

### 4.1 Parasitological indicators

As shown in Table 2, the highest prevalence was recorded using bile examination (16.78% ± 1.83; 51/310) followed by liver dissection (11.28% ± 1.17; 68/603) and coprology (9.12% ± 1.08; 55/603) (*p* = 0.015). Infection intensity recorded by bile examination was higher than by coprology, 1968.2 eggs/mL of bile (range: 26–18,600) and 9.05 eggs/g of faeces (range: 0.6–135.8) (*p* = 0.002), respectively. While infection intensity recorded by liver dissection was 25.21 flukes/liver (range: 1–195). Infections abundance estimated by bile examination and coprology were 323.80 eggs/mL of bile and 0.83 eggs/g of faeces, respectively (*p* = 0.003). While infection abundance recorded by liver dissection was 2.84 flukes/liver (Table 2).

**TABLE 2:** *Fasciola hepatica* parasitological infection indicators of sheep in Sejnane slaughterhouse, Northwest Tunisia.

		Liver dissection						Coprological examination						Bile examination					
Factor	Parameter	Positive/examined (% ± S.E)	*p* value	Infection intensity ± S.E	*p* value	Infection abundance ± S.E	*p* value	Positive/examined (% ± S.E)	*p* value	Infection intensity ± S.E	*p* value	Infection abundance ± S.E	*p* value	Positive/examined (% ± S.E)	*p* value	Infection intensity ± S.E	*p* value	Infection abundance ± S.E	*p* value
**Age** **(years)**	<1	49/498 (8.13 ± 1.22)	**0.01 6** ^*, a^	34.98 ± 5.34	0.86	3.44 ± 5.34	0.95	41/498 (6.80 ± 1.14)	0.2^a^	12.14 ± 1.58	0.42	1 ± 1.52	0.18	33/235 (10.65 ± 2.01)	0.22^a^	3041.33 ± 634.64	**0.02** ^*^	427.14 ± 610.91	**0.04** ^*^
	1.3	15/83 (2.49 ± 3.64)		75.667 ± 5.52		20.37 ± 5.52		12/83 (1.99 ± 3.41)		18.25 ± 1.17		5.96 ± 1.6		14/55 (4.52 ± 4.84)		5192.93 ± 620.08		1330.16 ± 496.48	
	>3	4/22 (0.66 ± 7.08)		387.25 ± 6.93		74 ± 6.93		2/22 (0.33 ± 5.64)		219.7 ± 2.03		22.04 ± 1.6		4/20 (1.29 ± 7.61)		17250.75 ± 489.89		3450.15 ± 489.89	
**Breed**	Cross‐bred	64/478 (10.61± 1.39)	**0.01** ^*, a^	26.781 ± 5.34	0.9	3.59 ± 5.34	0.2	48/478 (7.96 ± 1.26)	0.3^a^	10.37 ± 1.58	0.77	1.04 ± 1.52	0.15	41/222 (13.23 ± 2.24)	0.21^a^	2448.24 ± 626.53	0.09	452.15 ± 614.74	**0.05** ^*^
	Queue Fine	0/52		NA		NA		1/52 (0.17 ± 1.87)		4.4 ± 1.32		2.95 ± 0.95		3/52 (0.97 ± 3.06)		24295.67 ± 545.99		1930.08 ± 624.54	
	Noire de Thibar	4/69 (0.66 ± 2.66)		210.5 ± 8.05		24.64 ± 8.05		6/69 (1.00 ± 3.13)		79.67 ± 1.77		7.2 ± 1.52		7/34 (2.26 ± 5.88)		9801 ± 607.89		2152.91 ± 465.79	
	Barbarine	0/4		NA		NA		0/4		NA		NA		0/2		NA		NA	
**Gender**	Male	5/31 (0.83 ± 5.76)	0.4^a^	222 ± 7.46	0.71	54.68 ± 7.46	0.4	3/31 (0.50 ± 4.86)	0.9^a^	115.4 ± 2.36	0.6	15.79 ± 1.69	0.91	1/8 (0.32 ± 10.48)	0.79^a^	307 ± 0	0.45	12496.13 ± 589.07	0.8
	Female	63/572 (10.45 ± 1.19)		27.206 ± 5.34		3 ± 5.34		52/572 (8.62 ± 1.11)		9.57 ± 1.58		0.87 ± 1.52		50/302 (16.13 ± 1.86)		2007.56 ± 626.53		333.28 ± 610.91	
**Season**	Summer	43/293 (7.13 ± 1.82)	**0.015** ^a^	29.047 ± 7.70	0.61	4.26 ± 7.70	**0.008** ^*^	34/293 (5.64 ± 1.69)	0.0^a^	10.13 ± 2.18	0.19	1.18 ± 2.12	**0.04** ^*^	NA		NA		NA	
	Winter	25/310 (4.15 ± 1.44)		18.6 ± 3.03		1.5 ± 3.03		21/310 (3.48 ± 1.34)		7.3 ± 1.02		0.49 ± 0.93		51/310 (16.45 ± 1.83)		1968.2 ± 626.53		323.8 ± 610.91	
**Overall**		68/603 (11.28 ± 1.17)		25.206 ± 5.34		2.84 ± 5.34		55/603 (9.12 ± 1.08)		9.05 ± 1.58		0.83 ± 1.52		51/310 (16.45 ± 1.83)		1968.2 ± 626.53		323.8 ± 610.91	

*Note*: Values in bold are significant *p*‐values.

Abbreviations: NA, not applicable; S.E, standard error.

^a^ Fisher exact test.

* Statistically significant (*p* ≤ 0.05).

Kappa concordance coefficient between liver dissection/coprological examination, liver dissection/bile examination, and coprological examination/bile examination were 0.85, 0.58, and 0.55, respectively, which represents a moderate and almost perfect agreement between the two techniques used according to the interpretation of Cohen's Kappa values (Landis & Koch, 1977).

Liver dissection technique revealed that the highest *F*. *hepatica* infection prevalence occurred in sheep under 1 year old (8.13% ± 1.22; 49/498) compared to sheep between 1 and 3 years old (2.49% ± 3.64; 15/83) and over 3 years old (0.66% ± 7.08; 4/22) (*p* = 0.016). The infection prevalence was highest in cross‐bred sheep (10.61% ± 1.39; 64/478), then Noire de Thibar breed (0.66% ± 2.66; 4/69) (*p* = 0.01), Barbarine breed (0/4), and Queue Fine de l'Ouest breed (0/52) (*p* = 0.01). Moreover, infection prevalence was higher during summer (7.13% ± 1.82; 43/293) compared to winter (4.15% ± 1.44; 25/310) (*p* = 0.015) (Table 2).

For bile examination, only age presented a significant factor for *F*. *hepatica* infection intensity, the highest infection intensity was recorded in sheep over 3 years old (17,250.75 ± 489.89 eggs/mL of bile) compared to sheep under 1 year of age and sheep between 1 and 3 years old (3041.33 ± 634.64 and 5192.93 ± 620.08 eggs/mL of bile, respectively) (*p* = 0.02). The highest infection abundance was recorded in sheep over 3 years old (3450.15 ± 489.89 eggs/mL of bile) compared to sheep under 1 year of age and sheep aged between 1 and 3 years (427.14 ± 610.91 and 1330.16 ± 496.48 eggs/mL of bile, respectively) (*p* = 0.04). Similarly, the highest abundance was recorded in Noire de Thibar breed (2152.91 ± 465.79 eggs/mL of bile), then cross‐bred and Queue Fine de l'Ouest breed (452.15 ± 614.74 and 1930.08 ± 624.54 eggs/mL of bile, respectively) (*p* = 0.05) (Table 2).

Liver dissection revealed that the highest infection abundance was recorded in summer (4.26 ± 7.70 flukes/liver) compared to winter (1.5 ± 3.03 flukes/liver) (*p* = 0.008) (Table 2).

For coprological examination, only the season factor represented a significant difference in *F*. *hepatica* infection abundance compared to other risk factors (breed, gender, and age) where a higher infection abundance was recorded during summer compared to winter, 1.18 ± 2.12 and 0.49 ± 0.93 eggs/g of faeces, respectively (*p* = 0.04) (Table 2).

### 4.2 Association between *F. hepatica* and gastrointestinal parasites infection

Sheep were infected by six parasite groups: *Eimeria* spp., *Moniezia* spp., *Nematodirus* spp., *Strongyloides* spp., *Trichuris* spp., and other STE. There was no statistically significant difference in infection prevalence, intensity, and abundance of *Eimeria* spp., *Moniezia* spp., *Nematodirus* spp., *Strongyloides* spp., *Trichuris* spp., and other STE according to *F*. *hepatica* infection status in sheep (*p* > 0.05) (Table 3).

**TABLE 3:** Parasitological indicators of *Fasciola hepatica*‐infected and *Fasciola hepatica*‐non‐infected sheep by coprology technique in Sejnane region, Northwest Tunisia.

	Positive/examined (% ± S.E)			Infection intensity ± S.E			Infection abundance ± S.E		
	*F*. *hepatica*‐infected sheep	*F*. *hepatica*‐non‐infected sheep	*p* value	*F*. *hepatica‐*infected sheep	*F*. *hepatica*‐non‐infected sheep	*p* value	*F*. *hepatica* infected sheep	*F*. *hepatica* non infected sheep	*p* value
*Eimeria s*pp.	61/94 (64.89 ± 3.92)	337/502 (67.13 ± 1.04)	0.85	1209.84 ± 799.83	1300.89 ± 945.41	0.25	785.11 ± 790.36	873.31 ± 939.75	0.78
*Moniezia s*pp.	2/94 (2.13 ± 1.46)	59/502 (11.75 ± 1.3)	0.09^a^	NA	NA	NA	NA	NA	NA
*Nematodirus s*pp.	4/94 (4.26 ± 2)	19/502 (3.78 ± 0.82)	0.84^a^	25 ± 10.49	168.42 ± 14.01	0.08	4.26 ± 8.15	6.37 ± 12.27	0.85
*Strongyloides s*pp.	1/94 (1.06 ± 1.05)	4/502 (0.80 ± 0.39)	0.8	100 ± 0	100 ± 6.5	0.9	1.06 ± 2.11	0.8 ± 1.58	0.8
*Trichuris s*pp.	3/94 (3.19 ± 1.76)	17/502 (3.39 ± 0.78)	0.93^a^	133.33 ± 44.9	158.82 ± 14.11	0.71	4.26 ± 8.24	5.38 ± 10.39	0.9
Strongyle eggs	24/94 (25.53 ± 3.71)	146/502 (29.08 ± 1.64)	0.6^a^	254.17 ± 106.75	280.82 ± 116.25	0.85	64.89 ± 96.65	81.67 ± 115.84	0.51

Abbreviations: NA, not applicable; S.E, standard error.

^a^ Fisher exact test.

## 5 Hematological parameters

The overall prevalence of anaemia in *F*. *hepatica*‐infected sheep was higher (22.73% ± 4.47; 20/88) compared to *F*. *hepatica*‐non‐infected sheep (8.02% ± 1.25; 38/474) (*p* < 0.05). While there was no statistically significant difference in infection prevalences of anaemic‐infected and anaemic‐non‐infected sheep according to all risk factors (breed, gender, and age) (*p* > 0.05) (Table 4).

**TABLE 4:** Haematological indicators of *Fasciola hepatica* infection in anaemic sheep in Sejnane region, Northwest Tunisia.

		*F*. *hepatica*‐infected anaemic sheep		*F*. *hepatica*‐non‐infected anaemic sheep	
Factor	Parameter	Positive/examined (% ± S.E)	*p* value	Positive/examined (% ± S.E)	*p* value
Age (years)	>1	12/65 (13.64 ± 4.81)	0.12^a^	28/397 (5.91 ± 1.29)	0.28^a^
	1.3	8/18 (9.09 ± 11.71)		8/60 (1.69 ± 1.39)	
	<3	0/5		2/17 (0.42 ± 7.81)	
Breed	Cross‐bred	20/75 (22.73 ± 5.11)	0.19^a^	30/367 (6.33 ± 1.43)	0.31^a^
	Queue Fine de l'Ouest	0/3		1/49 (0.21 ± 2.02)	
	Noire de Thibar	0/10		7/56 (1.48 ± 4.42)	
	Barbarine	NA		0/2	
Gender	Male	0/5	0.28^a^	1/20 (0.21 ± 4.87)	0.63^a^
	Female	20/83 (22.73 ± 4.69)		37/454 (7.81 ± 1.28)	
Overall		20/88 (22.73 ± 4.47)		38/474 (8.02 ± 1.25)	**0.0003** ^*^ ^a^

*Note*: Values in bold are significant *p*‐values.

Abbreviations: NA, not applicable; S.E, standard error.

^a^ Fisher exact test.

* Statistically significant (*p* ≤ 0.05).

## 6 DISCUSSION

The present study provides updated information on fasciolosis infection in sheep from Sejnane region, Northwest Tunisia. To our knowledge, this is the first investigation of ovine fasciolosis infection in Tunisia using three diagnostic techniques (liver dissection, bile examination, and coprology) and an assessment of some infection risk factors. Similarly, it is the first evaluation of the association between *Fasciola* and gastrointestinal parasites infection and the status of anaemia in *Fasciola*‐infected and *Fasciola*‐non‐infected sheep.

The overall liver fluke infection prevalence in the current study was estimated to be 16.78% (51/310), 11.28% (68/603), and 9.12% (55/603) using bile examination, liver dissection, and coprology, respectively. These prevalences were lower than those reported by previous studies in sheep from different Tunisian endemic regions, where *post‐mortem* analyses revealed that 70% of tracer lambs were infected in northwest Tunisia (Akkari et al., 2011), and the serological prevalence was estimated to 44% in the southwest of the country (Ayadi et al., 1997; Hammami et al., 2005). Moreover, parasite burdens found in the present study varied between 0 and 195 flukes per liver. Akkari et al. (2011) found a wider range of parasite burdens in tracer lambs from Sejane region in 2005, ranging from 0 to 321. Difference in the prevalence of fascioliasis between Tunisian north and south could be explained by the fact that northern Tunisia has a Mediterranean climate, although it is semiarid in the south of the country. On the other hand, the difference in prevalence within the same region could be attributed to climate change during the recent years, which influences the development of the intermediate host and subsequently the development of the parasite. Moreover, the intensive use of anthelmintic drugs affects the infection spread (Abdulhakim & Addis, 2012; Mehmood et al., 2017).

In other African countries, prevalence rates in sheep appear to be low (0.19%–6.5%) (Mbaya et al., 2011; Mohamed, 2013; Mungube et al., 2006; Ouchene‐Khelifi et al., 2018). Fasciolosis was highly endemic, mainly in Western Europe, where the overall prevalences of *F*. *hepatica* infection in sheep farms did not exceed 16% (Rinaldi et al., 2015). In Asia and America, infection rates were slightly higher, reaching 40% (Acici et al., 2017; Aghayan et al., 2019; Arbabi et al., 2018; Carmona & Tort, 2017). These differences in *F*. *hepatica* prevalence rates can be attributed to geographical and climatic factors such as temperature, humidity, precipitation, atmospheric pressure, wind direction and speed, cloud cover, visibility, and many other atmospheric variables, which influence the growth of the intermediate hosts (*Galba* spp.) and subsequently the development of *F*. *hepatica* larval stages (Abdulhakim & Addis, 2012; Mas‐Coma et al., 2008; Mehmood et al., 2017; Qin et al., 2016; Selemetas et al., 2015).

Prevalences of *F*. *hepatica* infection estimated with three techniques were significantly different, where the highest prevalence was obtained by bile examination (16.78%) followed by liver dissection (11.28%), then coprology (9.12%) (*p* = 0.015). Discrepancy in infection prevalence using different techniques is due to the difference of targeted stage (eggs or adult parasites) and the matrices (either faeces or liver) (Simo et al., 2020). Our results are consistent with those of Simo et al. (2020) in Cameroun where the highest prevalence of *F*. *gigantica* in cattle was detected by bile examination compared to the dissection of the main bile ducts and coprology: 33%, 7%, and 6%, respectively. They were also similar to those reported by Chaouadi et al. (2019) in Algeria where microscopic examination of the bile detected more cases of bovine fasciolosis than liver inspection, and those recorded by Rapsch et al. (2006) in Switzerland where bile examination had the highest sensitivity (93%) compared to the other techniques.

These findings mentioned above recommended bile examination for fasciolosis monitoring in slaughterhouses. Consequently, false negatives would be reduced, and true positives increased. It also makes it possible to detect contaminated farms so that live animals can be treated later to reduce economic losses.

Moreover, according to the Kappa agreement test results, there was a significant agreement among the results of bile examination, liver dissection, and coprology (Kappa = 0.85; 0.58; 0.55, and *p* = 0.015). This finding highlights that methods with greater sensitivity could be used or a combination of several methods simultaneously could be applied to diagnose *Fasciola* infection in livestock (Amiri et al., 2021).

*Fasciola hepatica* infection prevalence was significantly higher in sheep under 1 year old (8.13% ± 1.22; 49/498; *p* = 0.016), which is in line with other studies (Muleke & Otachi, 2017; Oljira et al., 2022; Zgabeher et al., 2012). This result could be explained by the presence of sequential infections in pastures (District & Belete, 2017). *Fasciola hepatica* infection prevalence in sheep was significantly higher in summer (7.13 ± 1.82%; 43/293) (*p* = 0.015) compared to winter (4.15 ± 1.44%; 25/310); this finding was in conformity with other reports (Akkari et al., 2011; Jemli et al., 1991; Novobilský et al., 2014; Stuen & Ersdal, 2022). Likewise, similar results showed that bovine *F*. *hepatica* infection prevalences were significantly higher in summer (Ouchene‐Khelifi et al., 2018; Mpisana et al., 2022). The high rate of liver fluke infection during summer could be explained by the high exposure to *F*. *hepatica* metacercariae on pastures during spring, when the grass is green and the transhibernating snails are more infected, and that the prepatent period in sheep takes around 10–12 weeks, corresponding to the summer season when the eggs are excreted by the definitive hosts (Byrne et al., 2018). Using the three screening techniques, we did not find any difference of *F. hepatica* infection prevalence according to gender (*p* > 0.05). The similar trend was reported by Bayu et al. (2013) and Oljira et al. (2022).

We found no statistically significant difference in co‐infection by *F*. *hepatica* and gastrointestinal parasites (*Eimeria* spp., *Moniezia* spp., *Nematodirus* spp., *Strongyloides* spp., *Trichuris* spp., and other STE). May et al. (2022) obtained similar results in German sheep and cattle. In contrast, Bellet et al. (2016) and Springer et al. (2021) reported significant positive associations between co‐infection with *Ostertagia* spp. and *F*. *hepatica* in low herd‐level body condition in UK and German dairy cattle, respectively. Likewise, Cuervo et al. (2012) reported a significant positive association between *F*. *hepatica* and *Eimeria* spp., *Nematodirus* spp., and strongyle eggs in goats living in Argentina.

The absence of statistically significant relation in the present study could be due to a low age of studied sheep but also to the lower infection risk by gastro‐intestinal parasites compared to *F*. *hepatica* since animals are treated against these parasites compared with *Fasciola* because sheep owners are more aware of the infection risk by gastrointestinal parasites, whereas infection by *Fasciola* is of less significant importance to them. Therefore, it is important to establish an integrated control programme including both fasciolosis and the main gastrointestinal parasites.

The association between *F*. *hepatica* infection status and anaemia based on haematological parameters showed that the overall prevalence of *F*. *hepatica*‐infected anaemic sheep was higher (22.73% ± 4.47; 20/88) compared to *F*. *hepatica* non‐infected anaemic sheep (8.02% ± 1.25; 38/474) (*p* < 0.05). Our results agreed with those reported by various studies showing a severe anaemia in natural *F*. *hepatica*‐infected sheep (Fouda et al., 2013; Kahl et al., 2021; Matanović et al., 2007; Rojo‐Vázquez et al., 2012). Anaemia in *Fasciola* spp.‐infected animals could be attributed to the blood spoliation by adult flukes that was estimated between 0.2 and 0.5 mL per day per fluke and haemorrhage due to adolescaria migration in the liver parenchyma (Behm & Sangster, 1999).

## 7 CONCLUSION

The present study confirms that *F*. *hepatica* infection is highly prevalent in sheep from Sejnane region, Northwest Tunisia. Sheep infection by gastrointestinal parasites did not vary according to *Fasciola* infection. The detection of *F*. *hepatica* eggs in bile is the most appropriate method for *post‐mortem* investigations of fasciolosis prevalence. This should motivate the various stakeholders to step up and intensify the screening and control of fasciolosis in sheep as well as in other ruminants. Farmers also need to be made aware of the economic losses caused by these parasites in their animals.

## AUTHOR CONTRIBUTIONS

**Ines Hammami**: Investigation; methodology; data analyses; writing‐original draft. **Yosra Amdouni**: Investigation. **Rihab Romdhane**: Investigation. **Limam Sassi**: Investigation. **Nadia Farhat**: Investigation. **Mourad Rekik**: Funding acquisition. **Mohamed Gharbi**: Conceptualization; supervision; funding acquisition; writing—review and editing.

### ETHICS STATEMENT

The present study was performed in a certified slaughterhouse under the supervision of an officially certified veterinarian by the Tunisian state.

### PEER REVIEW

The peer review history for this article is available at https://publons.com/publon/10.1002/vms3.1575.

## Data Availability

The data that support the findings of this study are available from the corresponding author upon reasonable request.
